# GH/IGF-1 axis in a large cohort of ß-thalassemia major adult patients: a cross-sectional study

**DOI:** 10.1007/s40618-022-01780-z

**Published:** 2022-03-19

**Authors:** I. Gagliardi, R. Mungari, M. R. Gamberini, M. Fortini, F. Dassie, M. C. Putti, P. Maffei, L. Aliberti, M. Bondanelli, M. C. Zatelli, M. R. Ambrosio

**Affiliations:** 1 Section of Endocrinology, Geriatrics and Internal Medicine, Department of Medical Sciences, University of Ferrara, Via Fossato di Mortara 64/B, 44121 Ferrara, Italy; 2 Unit of Thalassaemia and Haemoglobinopathies Day Hospital, Regional HUB Centre, Department of Medicine, Azienda Ospedaliero Universitaria S. Anna, Cona, Ferrara Italy; 3 Department of Medicine (DIMED), Clinica Medica 3, University of Padua, Padua, Italy; 4 Dipartimento di Salute della Donna e del Bambino (SDB), Clinica Oncoematologica, Padua University Hospital, Padua, Italy

**Keywords:** Beta-thalassemia, Adult, Growth hormone, IGF-1, Growth hormone deficiency

## Abstract

**Purpose:**

GH deficit (GHD) could represent an endocrine issue in ß-Thalassemia Major (ßTM) patients. GH/IGF-1 axis has not been extensively explored in ßTM adults, so far. We aim to assess GHD and IGF-1 deficiency prevalence in ßTM adult population, focusing on the relationship with liver disease.

**Methods:**

Cross-sectional multi-centre study conducted on 81 adult ßTM patients (44 males, mean age 41 ± 8 years) on transfusion and chelation therapy. GHD was investigated by GHRH + arginine test. IGF-1 levels, routine biochemical exams, Fibroscan, Hepatic Magnetic Resonance Imaging (MRI) and pituitary MRI were collected.

**Results:**

Eighteen patients were affected by GHD and 63 were not (nGHD) according to GHRH + arginine test, while basal GH levels did not differ. GHD was associated with a higher BMI and a worse lipid profile (*p* < 0.05). No significant differences were observed regarding liver function between the two groups. Pituitary MRI scan was normal except for one case of empty sella. The 94.4% and 93.6% of GHD and nGHD, respectively, presented lower IGF-1 levels than the reference range, and mean IGF-1 SDS was significantly lower in GHD patients.

**Conclusion:**

GHD is frequent in adult ßTM patients and is associated with higher BMI and worse lipid profile. nGHD patients present lower IGF-1 levels as well. There was no relationship between IGF-1 levels and liver disease. Further, multicentric studies with larger cohorts and standardized diagnostic protocols are needed.

## Introduction

ß-Thalassemia Major (ßTM) is a hereditary haematological disease characterized by reduced or abolished synthesis of β-globin chains [1]. The resulting chronic anaemia needs chronic transfusion treatment with concentrated red blood cells that might expose patients to several iron-overload comorbidities. Iron-chelating therapy contributes to reduce iron excess tissue deposits [2]. Consequently, improvement in ßTM management has significantly increased patient lifespan. Therefore, new challenges are rising about long-term management of transfusion-dependent chronic comorbidities in adult ßTM patients, such as endocrine diseases. The most frequent reported endocrine comorbidities are represented by hypogonadism, short-stature, impaired glucose intolerance and diabetes, hypothyroidism, bone disease [3–8]. Growth Hormone deficiency (GHD) is a potential finding in ßTM children during assessment for short stature with a wide prevalence rate between 8 and 50% [2, 9], probably due to the adoption of different diagnostic criteria. Some Authors documented GHD in adult patients, with a prevalence ranging between 8 and 54% [9–18]. However, GH/IGF-1 axis has not been as extensively explored in adult as in younger patients so far [5, 6, 19]. GHD in adult ßTM subjects may negatively impact on cardiovascular system, bone structure, lipid metabolism, and quality of life [20, 21]. Furthermore, in adult ßTM subjects, the role of IGF-1 levels is controversial, being frequently reduced regardless of GHD presence [9]. The pathogenesis of GH-IGF-1 axis impairment is multifactorial. Hypoxia secondary to chronic anaemia and haemosiderosis could play a role. However, the chronic liver inflammation associated with hepatic fibrosis, viral infections (mainly B and C hepatitis viruses) and iron toxicity cannot be excluded, as well [21]. The involvement of hepatic protein synthesis might justify reduced IGF-1 levels [9], although the evidence is still sparse. The present cross-sectional multi-centre study aims to assess GH-IGF-1 axis activity in a large cohort of ßTM adult patients, evaluating GHD and IGF-1 deficiency prevalence. The relationship between IGF-1 plasma levels and liver disease has also been investigated.

## Materials and methods

### Patients

A cohort of adult ßTM patients on transfusion and chelation therapy was recruited among patients followed by the two Italian endocrinological outpatient clinics of Ferrara and Padua in collaboration with the Thalassemia Center of Ferrara Hospital. Exclusion criteria were: diagnosis of thalassemia intermedia; age < 18 years; HIV infection; severe hepatic, cardiac or renal failure; pregnancy and breastfeeding. This study has been specifically approved by the Local Ethics Committee (Comitato Etico Indipendente di Area Vasta Emilia Centro, CE-AVEC, at the Policlinico S.Orsola-Malpighi in Bologna) and authorized by the General Director of the Azienda Ospedaliero Universitaria in Ferrara (protocol number CE-AVEC 690/2018/Oss/AOUFe). In addition, all subjects read and signed the informed consent form before enrolling in the study.

At the time of observation, all patients were investigated for GHD by GHRH + arginine test [22], with basal fasting IGF-1 levels evaluation. Any previously detected pituitary deficit was well replaced when GHRH + arginine test was performed in keeping with the guidelines [23]. Routine biochemical exams were evaluated: haemoglobin, albumin, total proteins, fibrinogen, aspartate aminotransferase, alanine aminotransferase, alkaline phosphatase, γ-glutamyl transferase, total and direct bilirubin, ferritin, glucose and insulin levels. Prothrombin time and partial thromboplastin time were registered. For each patient, demographic, anthropometric and clinical data were collected. When available, results from Fibroscan and hepatic Magnetic Resonance Imaging (MRI) were registered. Finally, if GHD was diagnosed sellar MRI with gadolinium enhancement was performed.

### GHRH* +* arginine test procedure

The test was performed in a fasting condition in the morning before the routine transfusion. In each patient, an indwelling catheter was placed into a forearm. GH and IGF-1 basal levels were measured before the stimulation. Intravenous injection of a bolus of 1 μg/kg GHRH (Ferring Pharmaceuticals Ltd., Hoofddorp, The Netherlands) at time 0 and arginine (l-Arginine hydrochloride 100 ml, 30%) from time 0 to + 30 min were administrated afterwards. Blood samples for GH levels were collected at − 15, 0, 30, 45, 60, and 90 min after GHRH administration. Blood samples were collected in EDTA tubes and then immediately centrifuged at 4 °C. Serum was collected by aspiration and stored at − 20 °C until GH was assessed.

### Biochemical assays

IGF-1 levels were measured by chemiluminescence immunoassay (LIAISON® IGF-1, DiaSorin, Saluggia, Italy) after IGF-1 separation from IGFBPs by sample acidification. Intra and inter-assays coefficients of variation were lower than 4.6% and 8.5%, respectively. IGF-1 level was expressed as an absolute value in ng/ml and as standard deviation score (IGF-1 SDS) according the following formula: (log[IGF-1] + 0.00625 × age − 2.555)/0.104. IGF-1 SDS value lower than -1.88 were (3° percentile) was considered subnormal [9].

GH levels were assessed with chemiluminescence immunoassay (Access® Ultrasensitive hGH, Beckman Coulter, Fullerton, CA). Intra and inter-assays coefficients of variation were 3.3% and 6.1%, respectively. GHD was diagnosed if post-stimulation GH peak was < 11 μg/l for those with a BMI < 25 kg/m^2^, < 8 μg/l for a BMI of 25–30 kg/m^2^ and < 4.2 μg/l for those with a BMI > 30 kg/m^2^ [22, 24].

### Instrumental exams

FibroScan was performed to assess hepatic fibrosis measured in kPa and classified according to the METAVIR score: F0–F1 (kPa < 7.1); F2 (kPa 7.1–9.4); F3 (kPa 9.5–12.4); F4 (kPa > 12.5). Hepatic MRIs allowed to calculate liver iron concentration (LIC, mg/g) on T2* ("T2-star") sequences. LIC was derived from the formula: LIC = (25.4/T2*) + 0.202. LIC value was used to identify 4 different levels of iron concentration: absent (LIC < 3.2 mg/g), mild (LIC 3.2–7 mg/g), moderate (LIC 7–15 mg/g), severe (LIC > 15 mg/g).

### Statistical analysis

Categorical variables were presented as absolute values/percentages and Exact Fisher's test was used to compare the results. Continuous variables were described by mean values ± standard deviation (SD) and were compared using the Student's test in case of a normal distribution or using the Mann–Whitney *U* test in case of non-normal distribution. Normality was tested with the Shapiro–Wilk test. Statistical correlation between two variables was evaluated with Pearson's or with Spearman's correlation in case of a normal or a non-normal distribution, respectively. Logistic regression analysis was performed to evaluate the actual association of different covariates with GHD. Bonferroni's correction was applied when appropriate. *p* value < 0.05 was considered statistically significant.

## Results

The cohort of adult ßTM patients was composed of 81 subjects (44 males and 37 females) with a mean age of 41 ± 8 years (range 19–56 years). All patients presented a complete pubertal development (Table 1). They were blood transfused on average every 17 ± 4 days and they all took iron chelation therapy. Sixty-eight patients had previously contracted HCV infection that was still active in 27. Fibroscan was performed on 76 subjects and over half of them (64.5%) have a METAVIR stage F0–F1, indicating absent or low-grade fibrosis. In the remaining five patients, Fibroscan could not be performed since they presented physical characteristics that prevented Fibroscan use. In addition, 77 patients underwent a hepatic MRI. In four cases, MRI was not performed due to patients’ refusal.

**Table 1:** Patients anthropometric and clinical characteristics

	Total	GHD	nGHD
No. of patients (*n*, %)	81 (100%)	18 (22.2%)	63 (77.8%)
Age (years ± SD)	41 ± 8.29	41.08 ± 7.38	40.97 ± 8.59
Gender (M/F)	44/37	10-Aug	34/29
Weight (kg ± SD)	59.63 ± 7.86	64.02 ± 7.87*	58.38 ± 7.45*
Height (m)	1.61 ± 0.07	1.62 ± 0.7	1.60 ± 0.7
BMI (kg/m^2^ ± SD)	23.08 ± 2.8	24.34 ± 2.74*	22.72 ± 2.73*
Normal (*n*, %)	61 (75.3%)	11 (61.1%)	50 (79.4%)
Overweight (*n*, %)	19 (23.4%)	7 (38.9%)	12 (19%)
Obese (*n*, %)	1 (1.3%)	0 (0%)	1 (1.6%)
Transfusion frequency (day ± SD)	17 ± 3.86	17 ± 4.42	17 ± 3.73
Active HCV infection (*n*, %)	27 (33.3%)	6 (33.3%)	21 (33.3%)
Inactive HCV infection (*n*, %)	41 (50.7%)	8 (44.5%)	33 (52.4%)
No HCV infection (*n*, %)	13 (16%)	4 (22.2%)	9 (14.3%)
Fibrosis (kPa ± SD)	7.12 ± 3.81	6.8 ± 2.24	7.23 ± 4.17
F0–F1 (*n*, %)	49 (64.5%)	12 (70.6%)	37 (62.7%)
F2 (*n*, %)	15 (19.7%)	3 (17.6%)	12 (20.3%)
F3 (*n*, %)	10 (13.1%)	2 (11.8%)	8 (13.5%)
F4 (*n*, %)	2 (2.7%)	0 (0%)	2 (3.5%)
LIC (mg/g ± SD)	5.39 ± 7.11	7.13 ± 10.33	4.86 ± 5.8
Absent (*n*, %)	47 (61%)	12 (70.6%)	37 (62.7%)
Mild (*n*, %)	12 (15.6%)	3 (17.6%)	12 (20.3%)
Moderate (*n*, %)	8 (10.4%)	2 (11.8%)	8 (13.5%)
Severe (*n*, %)	10 (13%)	0 (0%)	2 (3.5%)
Total cholesterol (mg/dl ± SD)	116.66 ± 29.57	103.28 ± 27.37*	120.48 ± 29.26*
HDL	38.99 ± 16.11	30.86 ± 12.59*	41.31 ± 16.33*
LDL (calculated)	38.31 ± 22.4	50.08 ± 23.44*	60.66 ± 21.72*
Triglycerides	102.96 ± 55.77	123.59 ± 69.14	97.06 ± 50.43

*GHD* growth hormone deficiency, *nGHD* no growth hormone deficiency, *BMI* Body Mass Index, *HCV* hepatitis C virus, *LIC* liver iron concentration

*p < 0.05

GHRH + arginine test diagnosed 18 patients with GHD while 63 did not present this dysfunction (nGHD). GHD did not differ from nGHD patients in terms of age and sex distribution. GHD patients showed higher mean weight and BMI values (*p* < 0.05), while no significant differences were observed regarding transfusion and chelation therapy, active HCV infection disease, grade of hepatic fibrosis and iron saturation at Fibroscan and liver MRI, respectively (Table 1). Liver function biochemical parameters did not differ except for bilirubin levels that were significantly higher in GHD patients (2.73 ± 2.16 mg/dl vs. 1.72 ± 0.94 mg/dl, *p* < 0.05). The other biochemical analytes did not significantly differ between the two groups except for total cholesterol, HDL and LDL levels that were higher in GHD patients (Table 1). The prevalence of other pituitary hormonal impairment did not differ between the two groups. We assessed 2 cases of hypoadrenalism, 33 of hypothyroidism and 58 of hypogonadism which were adequately replaced at the moment of evaluation. When pituitary MRI was performed, we detected one empty sella and no cases of sellar masses. The 94.4% and 93.6% of GHD and nGHD patients, respectively, presented lower IGF-1 levels than the reference range, and mean IGF-1 SDS was significantly lower in GHD patients. Basal GH levels did not differ (Table 2). After logistic regression analysis, weight and IGF1 SDS were confirmed as significantly associated with GHD (*p* < 0.05). Finally, we found a negative correlation between GH peak and weight and between GH peak and BMI.

**Table 2:** GH/IGF-1 axis assessment

	Total	GHD	nGHD
Basal GH (µg/L ± SD)	0.99 ± 1.48	0.46 ± 0.47	1.15 ± 1.62
Peak GH (µg/L ± SD)	19.64 ± 12.15	6.84 ± 2.75*	23.29 ± 11.38*
IGF-1 SDS (SDS ± SD)	− 5.31 ± 2.38	− 6.61 ± 2.4*	− 4.94 ± 2.26*
Patient with normal IGF-1 levels (*n*, %)	5 (6.2%)	1 (5.5%)	4 (6.4%)
Patient with low IGF-1 levels (*n*, %)	76 (93.8%)	17 (94.4%)	59 (93.6%)

*p < 0.05

## Discussion

Our study contributes to characterize GH/IGF-1 axis activity in adult ßTM patients, where we confirm a consistent GHD prevalence. However, we also observed that in nGHD patients, IGF-1 levels tend to be lower than the reference range.

ßTM disease requires lifelong supportive treatment with blood transfusions, which might expose to several iron-overload comorbidities. Implementation with iron chelation therapy has prolonged the lifespan of ßTM patients reducing multisystemic iron toxicity impact, even if it has not been wholly erased [25]. Increased life expectancy has turned the attention towards the long-term management of transfusion-dependent chronic comorbidities such as cardiomyopathy and liver disease [26]. Furthermore, the interest in endocrine complications among adult patients is growing. The most frequent reported endocrine comorbidities are hypogonadism, short-stature, impaired glucose intolerance and diabetes, hypothyroidism, and bone disease [3–8]. A retrospective analysis conducted on a long-lived (45–60 years) selected cohort of adult ßTM transfusion-dependent patients revealed that more than 88% of subjects presented at least one endocrine comorbidity [8]. However, GH/IGF-1 axis has not been as extensively explored in the adult population [5, 6, 19], as in younger patients who are often tested for GHD during investigation for short stature to provide replacement therapy if needed [4, 21]. Considering that extensive studies addressing GH/IGF-1 axis function in adult ß-TM patients are very sparse (Table 3), we aim to investigate this issue in a large cohort of adult patients routinely undergoing endocrine evaluation at our centres. GHD was found in nearly a quarter of patients, and only in one case we found a pituitary morphological abnormality.

**Table 3:** Literature ßTM series tested for GHD

Author	Year	Patients (no)	Age range (years) or mean age (years ± SD)	Included patients	Provocative test	GHD diagnosis	Laboratory assay	GHD prevalence (%)
La Rosa et al.	2005	16	21–39 years	ßTM	GHRH + arginine test	GH peak < 9 μg/l	Chemiluminescent immunoassay	19%
Scacchi et al.	2007	94	18–50 years	69 βTM 25 βTI	GHRH + arginine test	Severe GHD GH peak < 9 μg/l Partial GHD GH: 9–16.5 μg/l	Chemiluminescent immunoassay	Severe GHD: 22.3% Partial GHD: 19.1%
Vidergor et al.	2007	16	29.3 ± 6.9	15 βTM 1 βTI	GHRH + arginine test	GH peak < 11.0 μg/l for BMI < 25 kg/m^2^ GH peak < 8.0 μg/l for BMI 25–30 kg/m^2^ GH peak < 4.1 μg/l for BMI > 30 kg/m^2^	Chemiluminescent immunoassay	25%
Scacchi et al.	2008	64	18 – 45 years	49 βTM 15 βTI	GHRH + arginine test	Severe GHD GH peak < 9 μg/l Partial GHD GH: 9–16.5 μg/l	Chemiluminescent immunoassay	Severe GHD: 25% Partial GHD: 17.1%
De Sanctis et al.	2011	32	25–53 years	ßTM	GST	GH peak < 3 μg/l	Chemiluminescent immunoassay	54.5%
Pincelli et al.	2011	25	26.6 ± 6.4 years	ßTM	GHRH + arginine test GHRH + pyridostigmine	GH peak < 9 μg/l to two GH provocative**	Chemiluminescent immunoassay	8%
Poggi et al.	2011	28	30 ± 6.2 years	ßTM	GHRH + arginine test	GH peak < 11.0 μg/l for BMI < 25 kg/m^2^ GH peak < 8.0 μg/l for BMI > 25 kg/m^2*^	Chemiluminescent immunoassay	32.1%
Soliman et al	2014	30	31.5 ± 7.2 years	ßTM	Clonidine test	GH peak < 7 μg/l	Immunoradiometric assay (IRMA)	40%
De Sanctis et al	2014	11	25–49 years	ßTM	GST	GH peak < 3 μg/l	Chemiluminescent immunoassay	54.5%
Scacchi et al	2016	68	28–58 years	132 βTM 7 βTI	GHRH + arginine test	GH peak < 9 μg/l	Chemiluminescent immunoassay	27.9%

*GHD* GH deficit, *GST* glucagon stimulation test, *TI* thalassaemia intermedia

*No patient with BMI > 26 kg/m^2^

**If GH peak was < 9 μg/l, GHRH + pyridostigmine test was performed

GHD aetiology might be due to chronic iron overload affecting hypothalamic and pituitary structures, even if literature data are still not conclusive [27]. Compared to nGHD patients, the GHD group presented significantly lower basal and peak GH levels, confirming GH secretion impairment. As a GHD consequence, patients presented higher BMI with a worse lipid profile. These data support the role of GH in influencing metabolism and body composition [28], which often are already compromised as a consequence of both ßTM itself and other endocrine comorbidities such as hypogonadism. Indeed, in our cohort, gonadal and thyroid axis were the most affected, as previously seen in other Italian series [6, 26]. However, they were all adequately replaced at the moment of this investigation. Increased BMI and dyslipidemia are two cardiovascular risk factors that may expose patients to cardiac disease, considering that ßTM patients might already present iron-induced cardiomyopathy.

Furthermore, GHD itself could affect cardiovascular function [28]. Campo et al. observed in a cohort of 21 ßTM patients a strong association between GHD and the presence of structural and functional cardiac dysfunction [20]. Moreover, GHD in ßTM was frequently found to be associated with bone disease [21]. Therefore, replacement therapy with rhGH should also be considered for adult patients with ßTM to optimize the management of other chronic comorbidities and improve patients' quality of life [21].

In our cohort, GHD patients represent almost a quarter of the whole sample (22%). Previous series describing adult cohorts of ßTM patients (≥ 18 years) reported a GHD prevalence ranging from 8 to 54% (Table 3) [9–18]. This wide variability in GHD prevalence could be explained by the different GHD diagnostic protocols depending on the time of evaluation (2005–2016). GHRH + arginine test has been the mostly used provocative test; however, different interpretation cut-offs were applied and sometimes a distinction between severe and partial GHD was adopted. The present study on GH/IGF-1 axis might be compared with that of Poggi et al. [15] and Vidergor et al. [11] because they referred to a BMI-adjusted GHD interpretation cut-offs, as suggested by the GHD guideline for the general population [22]. However, they analysed much smaller cohorts (28 and 16 patients), finding a slightly higher GHD prevalence (32% and 25%, respectively). Glucagon stimulation test was used only in two cohorts [13, 17] and Clonidine test in one study [16]. In one case the protocol consisted of a confirmation pyridostigmine test in case of GH peak < 9 μg/l at the previous GHRH + arginine test [14]. Chemiluminescent immunoassay was mainly used, except for one case using immunoradiometric assay (IRMA) [16].

We found that IGF-1 levels were lower than the reference range, even in nGHD group. Previously, iron-induced liver disease was considered as the leading cause of lower IGF-1 levels. Yassin et al. observed that adult ß-Thalassemia patients with higher LIC presented lower IGF-1 levels. Furthermore, they found a significant correlation between LIC, ferritin, and IGF-1 levels [29]. On the contrary, we did not observe any association between IGF-1 levels and hepatic function. This discrepancy could be explained considering that Yassin et al. [29] did not exclude patients with more severe hepatic conditions as we did. On the other hand, in agreement with Scacchi [9] and De Sanctis [17] results, we found IGF1 levels lower than reference range in nGHD patients, and no correlations were observed between ferritin/GH peaks and ferritin/IGF-I SDS. These data suggest that other mechanisms are involved in IGF-1 impaired secretion besides iron overload. However, Scacchi et al. included in their cohort a subgroup of patients with ß-Thalassemia intermedia who are less dependent on blood transfusions [9]. The confirmation of lower IGF-1 levels in adult ßTM patients could suggest a lower therapeutic target. Indeed, pursuing IGF-1 levels in the reference range of the general population during rGH replacement therapy might increase the risk of over-treatment and related adverse events in adult ßTM patients.

A strength of our study was the larger sample compared to the previous series and the cohort homogeneity due to the inclusion of only adult ßTM patients, all satisfactorily treated with transfusion and chelation therapy. However, a limitation is that Fibroscan and hepatic MRI were not available for each subject. Furthermore, sellar MRI was performed only if GHD was diagnosed, according to clinical practice management.

In conclusion, GHD is still a frequent finding in the adult ßTM population associated with higher BMI and worse lipid profile, further compromising patient health and quality of life. The nGHD population seems to present lower IGF-1 levels as well, without any relationship with liver disease. Therefore, replacement therapy with rhGH should also be considered for adult ßTM patients. However, it should be recognized that IGF-1 target levels might be lower than the non-ß-Thalassemia population. Other multicentric studies with larger cohorts and standardized protocols are needed to define GH/IGF-1 axis function and the real impact of GHD on ßTM comorbidities. Specific IGF-1 ßTM reference range levels could be helpful for tailored titration of rhGH therapy to avoid overtreatment.

## Data Availability

The data that support the findings of this study are available on request from the corresponding author. The data are not publicly available due to privacy or ethical restrictions.
